# Numerical Analyses of Fracture Mechanism of the Pelvic Ring during Side-Impact Load

**DOI:** 10.3390/ma15165734

**Published:** 2022-08-19

**Authors:** Tomasz Klekiel, Katarzyna Arkusz, Grzegorz Sławiński, Piotr Malesa, Romuald Będziński

**Affiliations:** 1 Faculty of Mechanical Engineering, University of Zielona Góra, Prof. Z. Szafrana 4 Street, 65-516 Zielona Góra, Poland; 2 Faculty of Mechanical Engineering, Military University of Technology, Gen. Sylwestra Kaliskiego Street 2, 00-908 Warsaw, Poland

**Keywords:** pelvic, bone structure, fracture bone mechanism, Johnson–Cook material model, LS-DYNA, impact load

## Abstract

The aim of this study is the analysis of the multiple pelvis fracture mechanism in side-impact dynamic load cases. The elaborated numerical model of a pelvis complex includes pelvic and sacral bones as well as soft tissues such as ligaments and cartilages. The bone has been modelled as a viscoelasticity material based on the Johnson–Cook model. The model parameters have been chosen based on the experimental data. The uniqueness of a presented approach refers to the selection of crack criteria for the bone. Thus, it was allowed to analyse the process of multiple fractures inside the pelvic bones. The analysis was evaluated for the model in which the deformation rate influences the bone material properties. As a result, the stress distributions inside particular bones were changed. It has been estimated that the results can vary by 50% or even more depending on the type of boundary conditions adopted. The second step of work was a numerical analysis of military vehicle subjected to an IED. An analysis of the impactor’s impact on the pelvis of the Hybrid ES-2RE mannequin was conducted. It was shown that the force in the pelvis exceeds the critical value by a factor of 10. The results of the numerical analysis were then used to validate the model of a military vehicle with a soldier. It was shown that for the adopted loading conditions, the critical value of the force in the pelvis was not exceeded.

## 1. Introduction

The validated model made possible an analysis of various load variants. The explicit algorithm for finite element method (FEM) is useful in model preparation. The models, among others, are used to model a fracture mechanism in the body, but the analysis quality depends on the material behaviour under impact load. As a result of a side impact, the passengers of a vehicle are usually injured in the body segment, such as the abdomen, chest, and head due to the high energy. Fractures are also associated with the pelvis. Using a numerical method, a better understanding of the bone fracture mechanism is possible. The body model with appropriate boundary conditions should be prepared and validated based on experimental data [1]. The energy transfer to passengers through the vehicle’s elements under a side-impact crash is typically analysed using FEM [2]. According to previous studies, the influence of the impulse loads acting on a passenger’s body during a traffic accident depends on the vehicle’s velocity during a collision [3]. The distribution of passengers in a vehicle, the type of a vehicle, as well as age, growth, and weight of passengers influence the type of injuries [4,5]. Similar factors also have an influence on soldiers travelling in a military vehicle.

The safety of a passenger during a side impact is an important issue. The force acting on the pelvis in the lateral direction generates a lateral compression of the pelvic ring, which causes a pelvic bone rotation inwards. This motion may cause damage to the pubic bone under a side impact. If the posterior cruciate ligaments remain intact, the internal pelvic rotation causes further fractures of the anterior sacrum part and the hip plate, known as a Malgaigne fracture [6]. If the loading is focused at the height of a pubic symphysis, the previously listed injuries are observed on the side opposite to the working force [7]. The mechanism of these fractures remains unclear and makes developing effective vehicle protection more difficult. The numerical analysis of the bone’s fracture mechanism in a pelvic ring indicates that the structures are the most susceptible to injury in the following order: first the right pubic ramus and ischium, next the left pubic ramus and ischium, finally fractions of the left iliac fossa and acetabulum [8]. The bending stress due to out-of-loading was analysed by Majumder et al. [9].

The numerical simulation of pelvic destruction requires a special approach in three basic aspects: modelling of multiple fragment fractures, soft tissue structures as energy dissipating elements, mechanical properties dependent on a bone deformation rate and different stiffness as a result of density distribution inside the bone. The external layer of the cortical bone has the maximum strength with ultimate stress of 135 MPa, and usually the highest stresses are concentrated in this layer [10]. The numerical analysis argued that the stresses are mainly concentrated in cortical bone with higher stiffness. The pelvic ring stiffness changes after bone fracture [1]. The set of the bones after fracture creates a different load distribution state, in which some part of the energy is dissipated, and a stiffness set is much smaller. The bone’s mechanical strength is strongly dependent on the deformation rate. The experiments performed by Martin et al. suggested that the bone reaction is greater for high impactor speed [11]. One of the assumptions of bone modelling is an approximation of a bone structure as a fibre-reinforced composite with different density inside and outside its geometry. The mean values of the Young modulus of the osteonal and interstitial bone can be assumed as 20 GPa [12,13], but the density distribution influences the differences of stiffness and stress concentration. In consequence, stress accumulation affects fracture propagation in the bone.

The discussion about property relations in the bone is helpful in the development of numerical models for the prediction of damage and strength of bones. The bone’s susceptibility to fracture is related to biomechanical properties, which depend on bone density, age, and health. Usually, the bone’s resistance to failure is defined by the ultimate strength resulting from maximum stress inside the structure existing under load [12]. However, the ultimate failure of a bone may be related to the maximum tensile or shear strain. The experimental investigations have shown that bone stiffness strongly depends on the deformation rate. The determination of a bone strength, among others, can be realised by the definition of a compressive yield strain for cancellous bone assumed as 0.7% [14] and the ultimate compressive strain as 1.5% [15]. The tensile strain in a sacrum is about 0.8%, but the ultimate compressive strain is 2.4% [16].

The bone is the most investigated material for which the experimental data are used to formulate material models defining the bone’s behaviour in FEM analyses. The experimental data were obtained for long bones during tension [17]. Very important information is the yield (ultimate) stress, and the maximal strain in a failure moment because, in practice, these parameters define bone strength [18]. The strain-stress relationship (SSR) shown that the bone is similar to the yielding materials, for which, after reaching the yield point, the strain increases but the force value not. The yield point is closely related to the deformation rate for the bony structures. The relationship between the ultimate stress and strain rate could define a stress failure criterion [19].

Thus, this paper presents the application of a yielding material model to simulate a multiple fragment fracture mechanism inside pelvic bone under side impact load. The Johnson–Cook (J–C) material model describes nonlinear strain hardening and strain-rate sensitivity. For this material model, the bone behaviour parameters were calculated based on the experimental stress-strain data for different deformation rates. The elaborated numerical model of the pelvic complex (PC) includes the most important details responsible for its rigidity and strength, including pelvic and sacral bones. The model also includes a soft tissue such as ligaments and cartilages. The bone material properties for the cortical layer were assumed based on the J–C model, for which the parameters were calculated based on experimental data presented in the literature. The obtained model was used to show the fracture process in a pelvic ring during side-impact. The proposed simulation method was used to investigate the interaction between the body and the seat, which influence the size of damages in the PC structure.

Finally, the results obtained were used to determine the risk of injury to a soldier in a military vehicle in the event of the detonation of an explosive device placed next to the vehicle. For this purpose, a vehicle model was used that was previously subjected to validation [20]. There was a Hybrid ES-2RE dummy in the vehicle, which made it possible to relate the results of numerical simulations of the impact of an explosive charge on a vehicle to a detailed pelvic model, which is the main purpose of this publication. The problem of numerical analysis in which of the human body is acting by the dynamic load is presented. The distribution stress in the selected segments of the body is helpful as an example in the safety analysis, but the right results from numerical analysis are strongly dependent on the modelling process. One of the important elements is the material and its representation in the model. Dynamic processes running in a short time cause some parts of the object to be deformed at different rates. The authors used the J–C material model to evaluate the model in the strain rate. The parameters of the J–C model were selected based on the bone tensile test. As a result, the damage to the pelvis in the given specific military accident are similar to the damage created in real conditions reported from battlefields.

## 2. Materials and Methods

A geometric model of the pelvic complex includes the skeletal, muscular, and ligament elements stabilising the pelvis. The model was elaborated based on the computed tomography images of a 25 year old patient. The simulations were realised by applying the impactor represented by force changed in time. In practice, the body mass is not rigidly fixed to the car and is not stiff, which suggests that the load force acting on the pelvis is less. The experimental data give that the minimal force value needed for pelvic damage is about 15 kN [9]. The load force in the simulation was defined as the function dependent on the time, changed from the initial value (15 kN) to zero. The decreasing force time influences the impact energy and the deformation rate. In the simulations, three different force decreasing times were selected. The vehicle inspection indicates that the door deflection caused the acting force on the passenger’s body, loading the femur and pelvic rim. A model was developed for reconstructing the passenger body load and the injury fracture mechanism in a simulated way.

### 2.1. Pelvic Bone Model Preparation

The geometry of the numerical model was obtained based on the tomography data. The sacroiliac joint was modelled as the frictional contact with a ratio of 0.01 [21]. The sacroiliac joint stabilisation was obtained by ligament stiffness. The pubic symphysis tissue was modelled as the set of one-dimensional links connecting two sides of the pelvic bones.

The stiffness of the links was calculated based on the data presented by Zaharie et al. [22]. The sacroiliac and pubic cartilages were defined as a solid isotropic material with Young modulus 11.6 and 10.1 MPa [10], respectively. The Poisson ratio for articular cartilage was assumed to be 0.49 [4]. The main pelvic ligaments are presented in Table 1.

**Figure 1:**
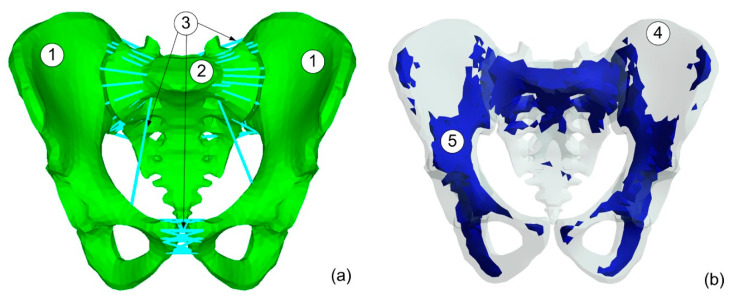
Model of pelvic ring with ligaments. (**a**) Pelvis model: 1—Pelvic bones, 2—Sacrum, 3—Ligaments, (**b**) Trabecular bone map: 4—cortical bone, 5—trabecular bone.

The designed model (Figure 1) includes the following ligaments: sacroiliac, pubic, inguinal, sacrospinous, and sacrotuberous, for which the material data were assumed based on experiments presented in [22]. The ligaments were prepared as the set of links for which the ligament stiffnesses were divided by the number of links representing the particular ligament. The pubic symphysis was modelled as the set of links. The stiffness of 120 N/mm was calculated based on the elastic module and mean cross-section area for the pubic symphysis. The cortical structures in the sacrum and pelvic bones were assumed to be the viscoelastic material dependent on the deformation rate modelled by the J–C material model. The mechanical properties of the ligaments were assumed based on Shi et al. work [23]. Table 1 includes the stiffness of the main ligaments used in the model [24]. These parameters were obtained in the test results for a female cadaver pelvis.

The bones in the model were divided into cortical and trabecular structures (Figure 1b). Both structures were modelled by solid elements in the common mesh differing only in their material properties. The trabecular bone was modelled as an isotropic elastic structure with the Young modulus equal to 400 MPa and Poisson ratio v = 0.2 [25].

### 2.2. Cortical Bone Characteristics

**Figure 2:**
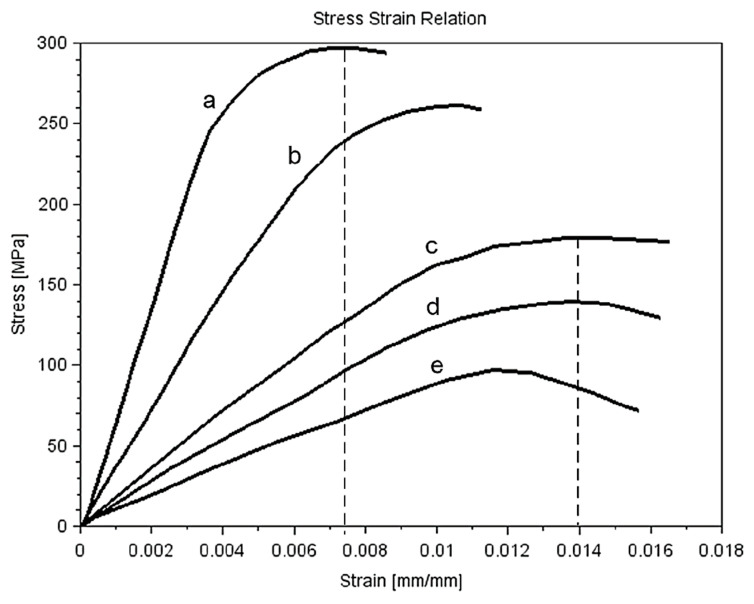
Relationship Stress-Strain for different strain ratio [26] (a) 1500 1/s, (b) 300 1/s, (c) 1 1/s, (d) 0.01 1/s, (e) 0.001 1/s.

The mechanical properties of the cortical bone were selected based on the J-C model used by Alam et al. [12]. The material model behaviour was compared with the SSR for the human femur bone. For comparison, the geometry of a typical tensile test specimen was prepared. The mechanical model property was investigated as a result of tension experiments calculated for the typical specimens used in a tensile test. Figure 2 presents the SSR for different deformation rates given by Viano et al. [26]. Curves (a) and (b) described cortical bone strength for deformation rates 300 and 1500 1/s, respectively. Next, curves c, d, and e present the SSR for lower deformation rates. These parameters were obtained as a result of testing a female cadaver pelvis. The ultimate stress is between 250 and 300 MPa. The ultimate strain for the presented data is in the range of 0.0072 to 0.014 mm/mm. The results suggested that, if the deformation rate increased, the Young modulus generally increased, but the yield strain decreased. Morgan and Keaveny [14] investigated the relationship between yield stress and density. The results suggested that the yield stress for different places has changed by 20% for trabecular bone.

Majumder et al. considered the trabecular and cortical bone as a bilinear elastoplastic model [16]. The experimental data also suggested that the tensile strain is more than the compressive strain. The mean strain value for the sacrum is equal to 1.6% and is shown in Figure 2. The J–C model described the yield point related to the strain rate according to equation [27]:

(1) $$\sigma {\left(\varepsilon \right)}_{yeld}=\left(A+G\varepsilon^{n}\right)\left(1+Cln\left(\frac{\dot{\varepsilon }}{\dot{\varepsilon_{0}}}\right)\right)$$

where *σ* is the equivalent stress and ε is the equivalent plastic strain.

The J–C material constraints are *A*, *B*, n, and *C*. *A* is the yield stress of the material under reference, B is the strain hardening constant, n is the strain hardening coefficient, and *C* is the strengthening coefficient of strain rate. Equation (1) does not include the member dependent on temperature. The material model was verified by the simple tension test for different deformation rates. (J–C) model of cortical bone was used for cutting simulation by Alam et al. [12]. The parameters for material in case I were *A* = 100 MPa, B = 51 MPa, *C* = 0.03 and n = 0.08. The bulk modulus for the cortical bone was assumed as 15 GPa, and the shear modulus was selected as 3.3 GPa [28].

**Figure 3:**
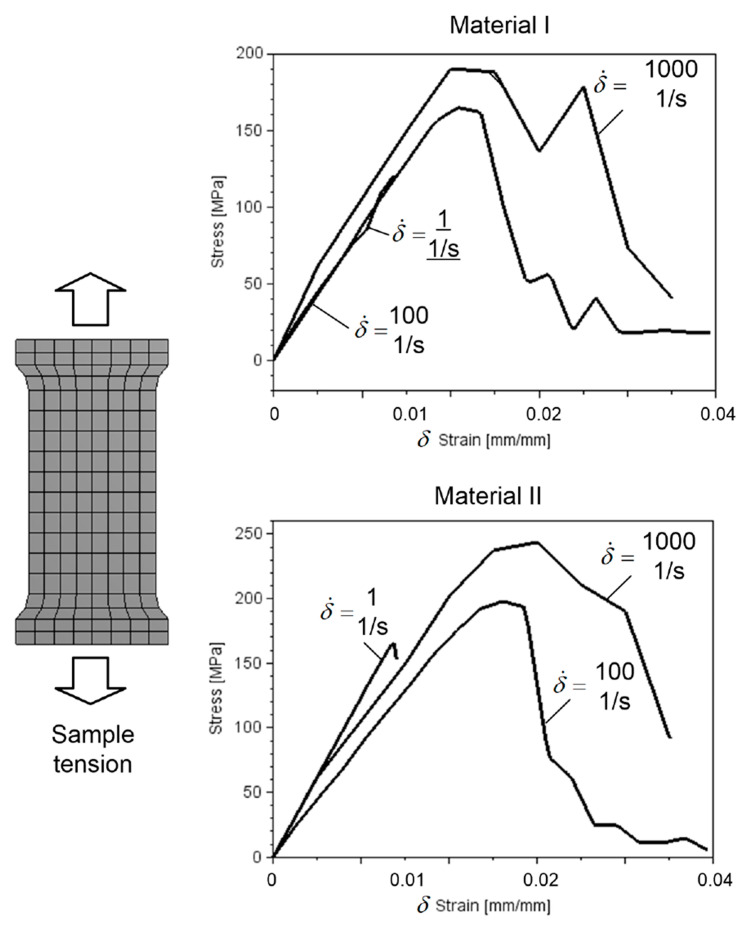
The Strain-Stress relationship (SSR) for two selected material models [26].

According to Equation (1), the *C* parameter is important in defining a SSR for different deformation rates. The analysis made by Alam et al. [12] concerns on cutting process for which the specimens were tested between 0.00001 1/s and 1 1/s strain rate. The cortical bone parameters for the J–C material model proposed by Alam et al. were selected for low deformation rates. The deformation of pelvic bones during side impact calculated during hitting is much higher. The parameters of cortical bone for both material cases are shown in Table 2 which include the parameters of the J–C model used in this analysis. The first line in the table includes the parameters assumed by the Alam et al. [12]. The parameters in the second line were taken from the numerical analysis for the model presented in Figure 3. The second parameters of the J–C material model were selected based on the Stress-Strain relationship given by Viano [26]. The material parameters for material II were selected as a result of numerical analysis. The fitting process relied on comparing the results given by Viano with results obtained from the numerical tensile test for the sample model presented in Figure 3, and was obtained based on the solution of the simple optimisation problem without any constraints, in which the purpose function was defined as the mean differential between the Stress-Strain curve for the material from experiment described in [26] and the curve resulting from the numerical investigation. For the next step of the investigation the material model parameters for the minimum value of the purpose function was used. The error of fitting was omitted.

The strain rate during impact is usually higher. It is suggested that the parameters in the model proposed by Alam et al. are not enough for higher deformation speeds. Figure 3 presents the stress-strain relationship analysed for two material models.

The numerical experiments were calculated for three deformation rates. Compared to the result presented in Figure 3 [26], the material II behaviour is similar to the bone tensile test presented by Viano.

### 2.3. Boundary Conditions

**Figure 4:**
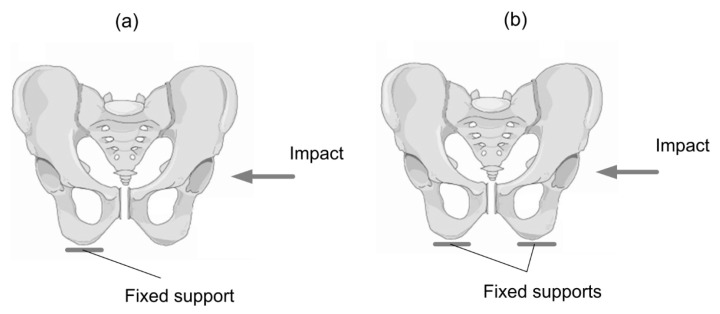
Two support configurations: (**a**) support on one pelvis bone on the opposite side than the impact support case I, (**b**) support on two pelvis bones support case II.

In the sitting position, a passenger inside a vehicle has contact between buttock tissue and a seat. The interaction is mostly dependent on the inertia force and the body position. Figure 4 presents two cases of sitting positions.

The first position (Figure 4a) suggests that the pelvic support is placed only on the pelvic bone fixed in the ischium opposite the impact. In the support case II, a seat is adjusted to the body shape, and both ischium bones are fixed (Figure 4b).

In most cases, a seat during sitting is deformed; consequently, any body movements related to the seat are difficult or impossible. During the seat design process, the emphasis is mainly placed on comfort, primarily associated with physiological and biomechanical factors. This is increasingly important because people spend the most time in this position. The study indicates that in order to obtain safety, a seat must be fixed to a body [29,30]. To achieve these priorities, immobilisation as fixed support was selected. The main limitations in the model are the result of boundary conditions in which the pubic arch is fixed to the seat. The model does not extend to the situation in which the body can move on the seat balanced of the reactions creating from the belts. In the paper the model includes two situations: body supported on one or two pubic arches, which is the situation that most often exists in the military accidents.

The load condition was represented by the maximal force acting on the PC. A person sitting in a car during a side impact is mainly loaded by a door deformation. The peak force value is dependent on the factors such as vehicle speed, body stiffness, shape, and mechanical properties of the seat. In this paper, the force value 15 kN was assumed as an average value of peak forces in the experimental tests presented in [16,31,32].

Significantly shorter times of impact peak are observed for loading generated by the blast wave propagation [33,34,35,36]. Based on the experimental and numerical data, the impulse period was assumed as the force 15 kN converged to 0 in three other impulse periods times 1 ms, 2 ms, and 4 ms, respectively. This solution involves the changes in the total velocity measured in the impacted place.

### 2.4. Validation Process

**Figure 5:**
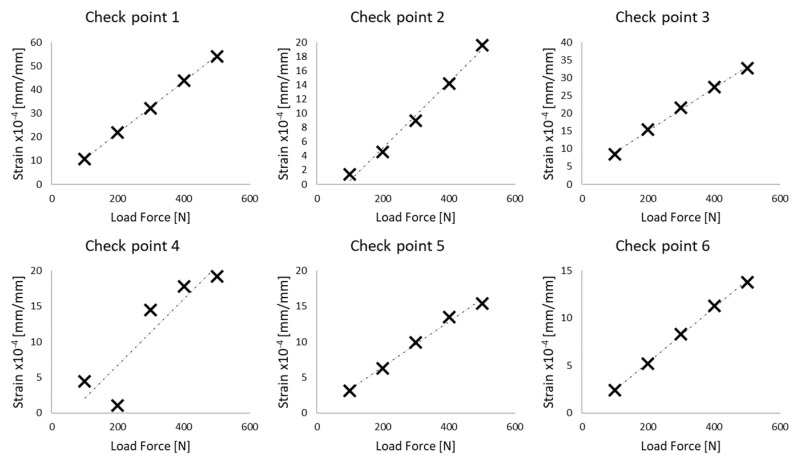
Validation of elaborated FE model for six points [25].

The validation process was similar to the method proposed by Hu et al. and was used successfully for a pelvic numerical model elaborated by Arkusz et al. [37]. Figure 5 shows the comparison data obtained for the presented PC model acting by the side force in the range from 100–600 N. For six different checkpoints placed on the pelvic bone, the differences between results obtained from the model and data given by Hu et al. [25] were calculated. The results are presented in Figure 5. The maximal error for the model was less than 7%. The results were similar for both analysed materials. In Figure 5, the lines represent the result for the elaborated model, and the points indicated by a cross come from the data presented by Hu et al.

### 2.5. Results and Discussion

**Figure 6:**
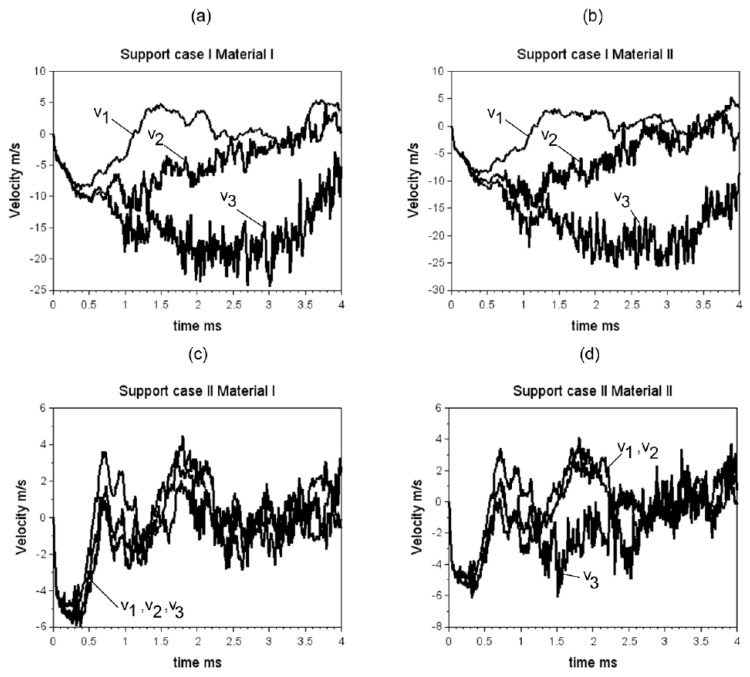
Velocity for both materials presented in Table 2 and two selected support cases presented in Figure 4: (**a**) Support case I and material I, (**b**) Support case I and material II, (**c**) Support case II and material I, (**d**) Support case II and material II.

The numerical experiments were planned in four steps. The first step focused on checking how the impact generated by the load force influences the velocity for the bone structure. All calculations were realised in the Explicit Dynamic module as part of Ansys C software. The next two steps are referred to the damage analysis of the bone for two support cases. The experiments have been done for the material II based on the J–C model and are presented in Table 2. The last step is a correlation of analyses carried out during the first three steps and another simulation using a vehicle and hybrid ES-2RE dummy for side impacts. Figure 6 presents the average velocity in a hip joint acetabulum after applying the force for a different impulse period time.

The impact force acting on the analysed model converged from 15 kN to 0 kN in 1 ms, generating the maximal deformation velocity 8 m/s. If the force is converged to 0 kN, in longer time the impact energy is greater, and the velocity measured in a hip joint acetabulum is increased to higher than 20 m/s. For the support case II the deformation velocity was smaller and similar independent to the load impulse characteristic. The results presented in Figure 6c are not dependent on material properties. Generally, the changes in the material model parameters, in reaction to the deformation rate, have not influenced the deformations in the force acting place.

### 2.6. Bone Fracture Analysis

**Figure 7:**
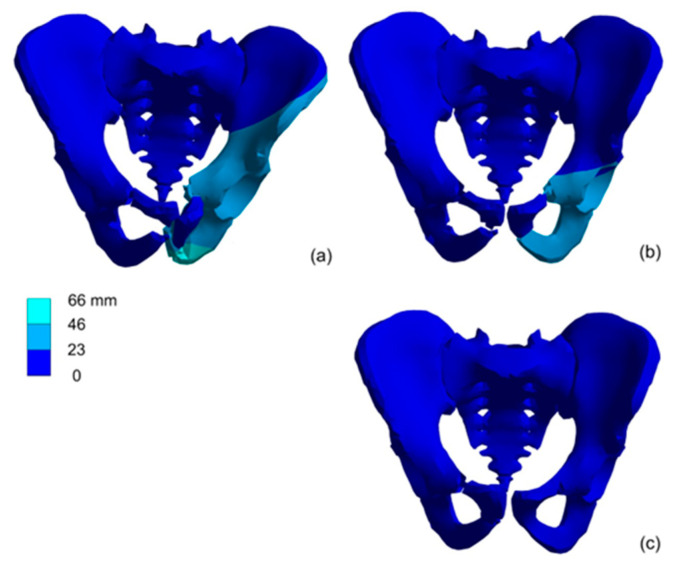
Displacements in the pelvis model for first support case for load impulse period time: (**a**) 4 ms, (**b**) 2 ms, (**c**) 1 ms.

The fracture mechanism was investigated for two support cases and for selected materials. The cortical bone called material II was defined by the parameters from Table 2. Figure 7 presents the maximal pelvis deformations presented at the moment of maximal load and on the same real scale. For the first variant of boundary conditions, a maximum displacement of 66 mm was obtained.

**Figure 8:**
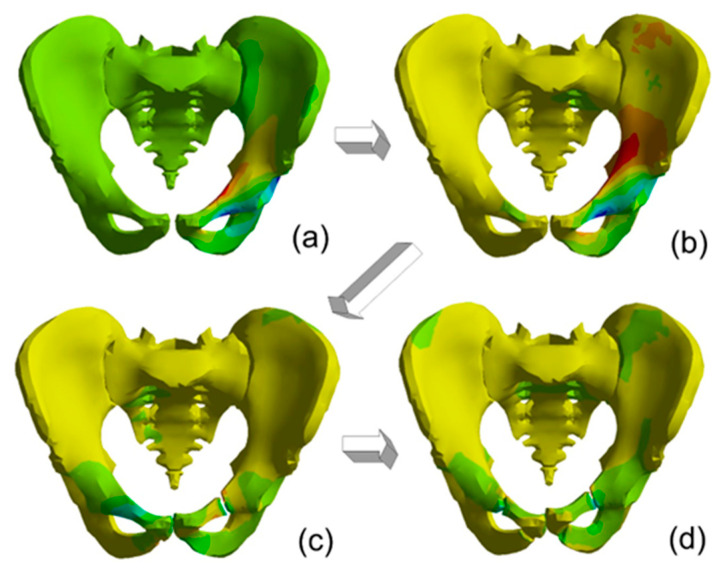
The steps in pelvic fracture for first support case: (**a**) below yield stresses in cortical bone, (**b**) the advantage of compressive forces in left superior pubic ramus, (**c**) Failure of the cortical bone forces in left superior pubic ramus, (**d**) Failure of the cortical bone forces in right superior pubic ramus and the left interior ramus.

For low deformation rates, the injuries inside the bones existed on the pelvic bone opposite to the load side. The peak force of 15 kN decreasing to 0 in 1 ms accelerate the bone to about 8 m/s. Figure 8 presents the progress in pelvic fractures under load for the I load case. The arrows show the next damage states in time.

For the maximal deformation rate, the failure of the bone was indicated as the stem of the left pubic bone, the right superior pubic ramus, and the left interior ramus (Figure 8). In each case, the pubic symphysis deformations were larger than the ultimate stress calculated by Li et al. [38]. The maximal strain rates exceeded 2620 1/s for load period time 1 ms and 2970 1/s for load period time 4 ms.

**Figure 9:**
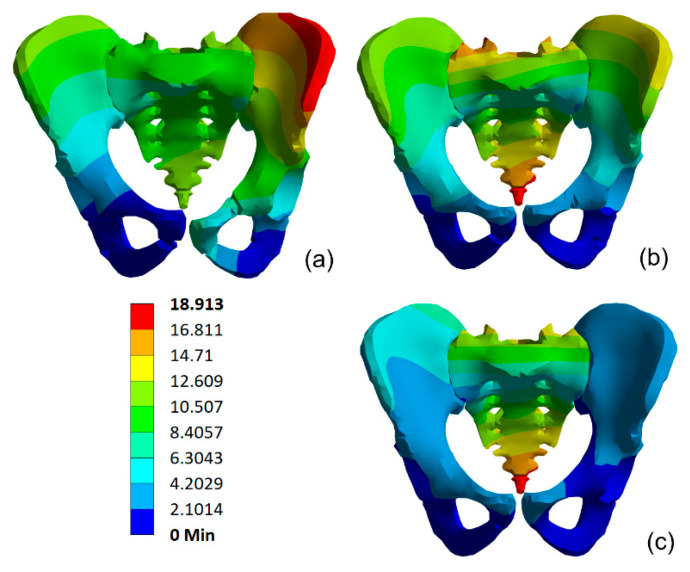
Displacements in the pelvis model for second support case for load impulse period time: (**a**) 4 ms, (**b**) 2 ms, (**c**) 1 ms.

For the second support case, the maximal deformations are smaller, and the injuries created under loading are similar for different load cases. For load case III, the pelvis is not destroyed (Figure 9). The double support of pelvic bones suggested that the damages and bone depositions are smaller. The loading velocity changes the deformation map, mainly in the pelvic bones.

For the second support case, the deformation rates are at least 50% smaller (Figure 9). It is possible because the pelvic ring is stiffer, and as a result the reaction force on the impact is higher but the deformation rate is lower. For the second variant of the boundary conditions, a maximum displacement of 18.913 mm was reached.

**Figure 10:**
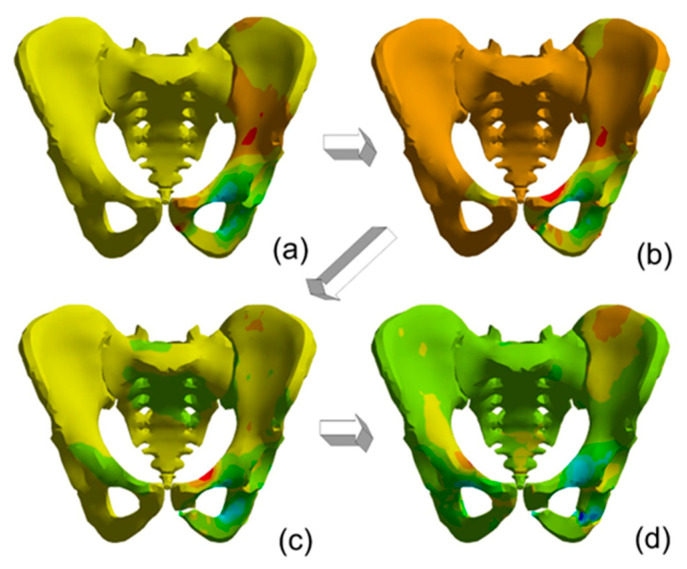
The steps in pelvic fracture for second support type and load impulse period time 1 ms. Map of normal stresses in next steps: (**a**) below yield stresses in cortical bone, (**b**) the advantage of compressive forces in left superior pubic ramus, (**c**) Failure of the cortical bone forces in left superior pubic ramus, (**d**) Failure of the cortical bone forces in right superior pubic ramus and the left interior ramus.

Figure 10 presents the progress in pelvic fractures under load for the II load case. The arrows show the next damage states in time.

The maps of normal stresses suggested that a seat is better adjusted to a body. The results for this support case indicate less injury. The maximum strain rate for load period time 1 ms is 1320 1/s but for load period 4 ms is 2220 1/s. The differences in strain rates suggest that, for the typical impact loads occurring during a crash with an impactor velocity of 10 m/s, the body support and the contact between a body and a seat have an important role in the risk of an injury, but for higher velocity this influence is not considerable.

## 3. Analysis of Risk of Pelvic Injuries Caused by IED

**Figure 11:**
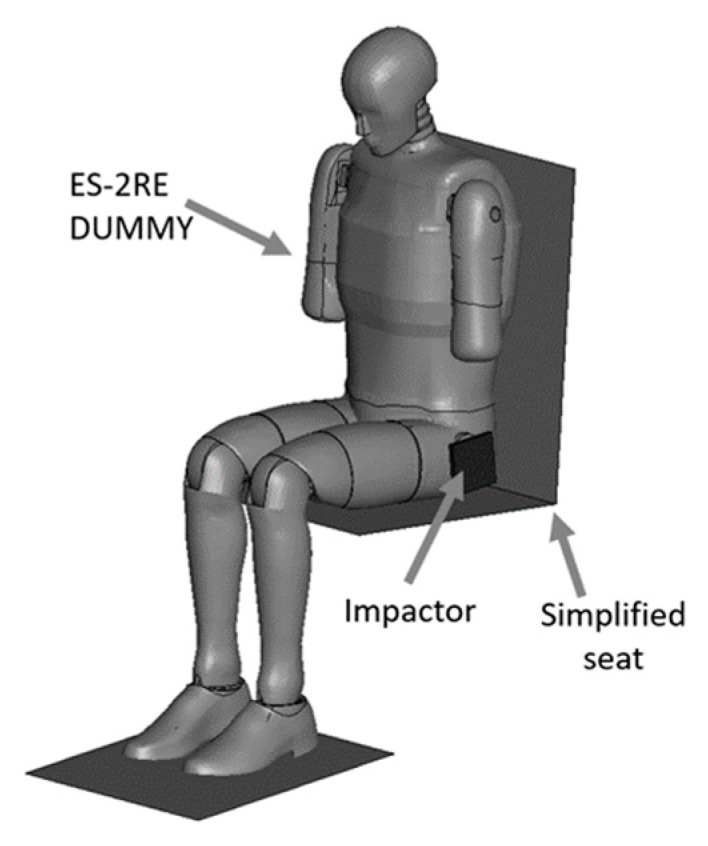
Modelled measurement station consisting of a dummy, seat and impactor.

The aim of the next stage of the study was to analyse the risk of a possible pelvic injury in a soldier in a vehicle in the event of a pressure wave applied to his structure from the explosion of a charge placed next to the vehicle. For numerical analyses carried out in the LS-Dyna environment, a Hybrid ES-2RE dummy model was used, seated on a stiff chair, which was then subjected to the impact of an impactor. A view of the prepared model is shown in Figure 11.

According to the approach of the authors of the publication [16], the mass of the impactor was 12 kg and its initial velocity was equal to 13.54 m/s. A numerical analysis with a length of 50 ms was performed, during which the contact force was measured. The sequence of shots from the course of the impact is presented in the Table 3.

**Figure 12:**
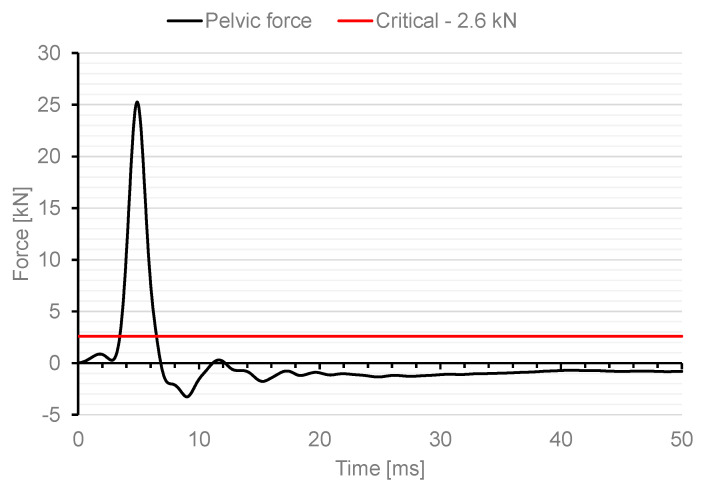
Characteristics of force change in a dummy pelvis after impact with an impactor.

As a result of the analysis, the impact force resulting from the contact of the impactor and the dummy with a value of 13 kN was measured. In the case of military tests, one of the normative documents defining the test conditions is the document AEP-55 Procedures for Evaluating the Protection Level of Armoured Vehicles. The document specifies, for example, critical values for individual parts of the soldier’s body for which there is a risk of injury. In the case of the pelvis, this is pelvis force, which determines the peak value of the lateral force measured on the pubic symphysis (pelvis). The maximum value of the pubic force is specified as 2.6 kN. Figure 12 shows the characteristics of the change in pelvic force versus time for the first 50 ms during the dummy load analysis with use of an impactor.

The maximum force in the dummy’s pelvis of 25.3 kN was recorded. This means that the critical value specified in the AEP-55 document was exceeded by a factor of 10.

**Figure 13:**
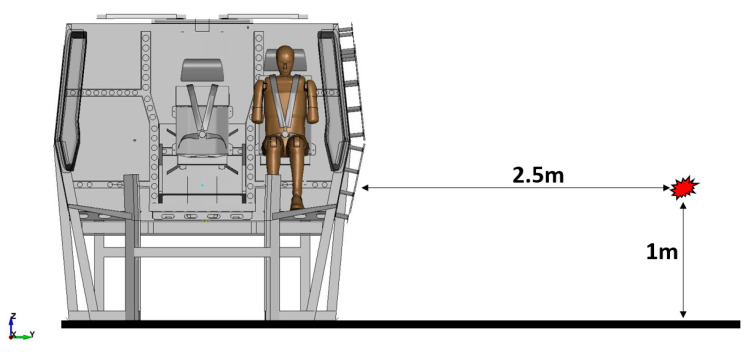
Way of placing the explosive towards the vehicle for side explosion.

For the model of the vehicle with the dummy in the passenger seat, which was described in more detail in the publication [20], an analogous measurement of the force in the dummy’s pelvis was carried out. In this case, the vehicle was loaded with a pressure wave from an explosion of a 15 kg TNT equivalent charge, according to the diagram shown in Figure 13 below.

**Figure 14:**
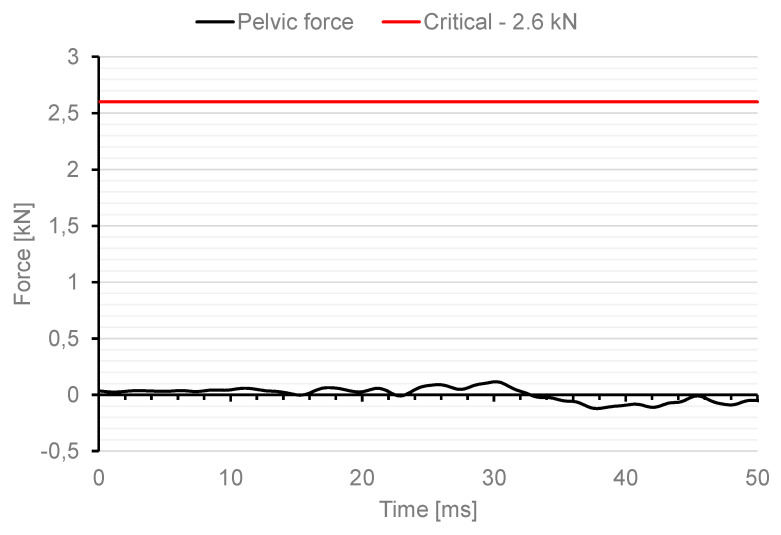
Characteristics of force change in a dummy pelvis after blast with use of 15 kg TNT charge.

The conditions of the analysis were performed in a manner analogous to the experimental tests described in the publication [20]. For the prepared variant of loading the vehicle with the pressure wave from the explosion of a 15 kg TNT charge, the maximum value of the force in the dummy’s pelvis was 0.116 kN and the minimum was −0.123 kN. Under the given conditions of numerical analysis, there is no contact between the dummy’s pelvis and the structural elements inside the vehicle. The results obtained in Figure 14 are much below the critical value specified in AEP-55.

## 4. Conclusions

In conclusion, this study aimed to develop the multi-fracture mechanism in the pelvis ring under side impact load using numerical analysis. An innovative feature of this study has been focused on bone behaviour when the strain rate depends on strength. Based on computed tomography, the developed model of the pelvic complex was characterised by considering all bone structures and ligaments. Moreover, the viscoelastic deformation of the pelvic complex was included in the modelling, which makes the developed model one of the most accurate so far.

The obtained results made it possible to determine the order of damage in the pelvic structure and the propagation of stresses depending on the working impactor and the sitting position. The changes in the force values generate different velocities in the acting force place. As a result, the bones are stressed at different deformation rates. The displacements are lower than the simple seats in which the pelvis is supported by the one side opposite to the load source. The displacements in the short time generate high deformation rates, which are the main source of high-stress concentration and bone fractures. As the cortical bone fracture criteria, the ultimate strain value equal to 1.5% was selected.

In a numerical analysis of the Hybrid ES-2RE dummy load using a 12 kg impactor with an initial velocity of 13.54 m/s, a force in the dummy’s pelvis of 25.3 kN was measured, which exceeds the allowable force by a factor of 10.

For the case of loading a military vehicle with a pressure wave resulting from the explosion of a charge of 15 kg TNT, the maximum value of the force in the dummy’s pelvis was measured to be 0.116 kN and the minimum value of −0.123 kN. Both values are much below the critical value, according to AEP-55. Analysing the results, it can be concluded that for the adopted loading conditions there is no risk of pelvic bone fracture.

## Figures and Tables

**Table 1:** Main pelvic ligaments included in the FEM model [22].

Ligament	Stiffness [N/mm]
Anterior sacroiliac ligament	700
Sacroiliac interosseous ligament	2800
Long posterior sacroiliac ligament	1000
Short posterior sacroiliac ligament	400
Sacrospinous ligament	1400
Sacrotuberous ligament	1500
Superior pubic ligaments	500
Arcuate pubic ligaments	500

**Table 2:** J–C model parameters for bone.

Material Case	*A*	*B*	*C*	n
Material I	50	101	0.03	0.08
Material II	50	101	0.15	0.08

**Table 3:** A sequence of photos from the stages of numerical analysis of the impactor’s impact on the dummy.

T = 0 ms	T = 10 ms	T = 20 ms
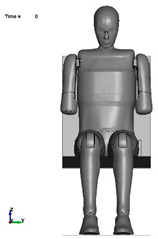	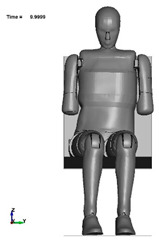	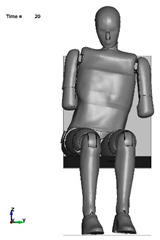
T = 30 ms	T = 40 ms	T = 50 ms
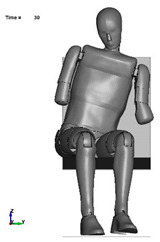	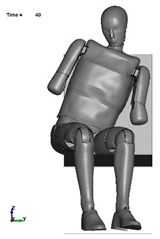	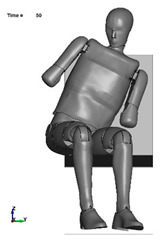

## Data Availability

The datasets presented in this article are not readily available because due to the military nature of the project “AFGAN” data are partially non-public. Requests to access the datasets should be directed to grzegorz.slawinski@wat.edu.pl.
